# A Prospective and Comparative Study to Explore the Effects of Platelet‐Rich Plasma in Hair Transplantation for Patients With Androgenetic Alopecia

**DOI:** 10.1111/jocd.16665

**Published:** 2024-11-18

**Authors:** Ping Xue, Lei Guo, E. Dang, Wenjie Dou, Xianhui Zeng, Xing Fan, Qing Yang

**Affiliations:** ^1^ Department of Plastic and Reconstructive Surgery, Xijing Hospital Fourth Military Medical University Xi'an Shaanxi People's Republic of China; ^2^ Department of Blood Transfusion, Xijing Hospital Fourth Military Medical University Xi'an Shaanxi People's Republic of China

## Abstract

**Background:**

Androgenetic alopecia (AGA) is the most prevalent type of hair loss. Traditionally, treatment for AGA has primarily involved the topical application of minoxidil in conjunction with oral finasteride or spironolactone. Recently, platelet‐rich plasma (PRP) has emerged as a significant focus of research in hair loss treatment. However, many studies on PRP‐assisted hair transplantation have encountered various limitations.

**Objective:**

This study aims to conduct a prospective, comparative clinical investigation to evaluate the therapeutic effects of combining PRP with minoxidil and finasteride/spironolactone as adjuncts to hair transplantation.

**Method:**

From August 2019 to December 2022, we enrolled 30 patients with AGA in the study, randomly assigning them to an experimental group and a control group. The experimental group received drug therapy alongside hair transplantation and underwent PRP injections, whereas the control group received only drug therapy to assist with hair transplantation.

**Results:**

Prior to surgery, no significant differences in baseline data were observed between the two groups. Following treatment, the experimental group demonstrated significantly improved follicle survival rates, follicle growth rates, and hair strength compared with the control group.

**Conclusion:**

This prospective, comparative clinical study demonstrated that the application of PRP in conjunction with pharmacological support during FUE treatment for AGA resulted in improved follicle survival rates, hair growth rates, and hair strength.

## 1 Introduction

Hair loss is a prevalent condition, affecting over 210 million people globally. By the age of 70, up to 80% of individuals may experience various forms of hair loss. The incidence of hair loss is higher in men than in women [1]. Although hair loss is not a malignant condition, it can significantly impair the quality of life and potentially lead to anxiety or depression [2]. Thus, diagnosing and treating hair loss has been a focus in both plastic surgery and dermatology. Among the different types of hair loss, androgenetic alopecia (AGA) is the most common [3]. It is a nonscarring alopecia primarily influenced by genetic factors [4]. AGA is characterized by a shortened anagen phase and the gradual transformation of terminal hairs into vellus hairs [5]. In men, AGA typically presents as a receding hairline and thinning of the hair on the crown. In women, it manifests as reduced hair density and finer hair diameter on the top of the head, whereas the hairline at the forehead usually remains unaffected [6].

For AGA, finasteride (for men only) and minoxidil are the only FDA‐approved treatments and have been traditional approaches for many years. However, because of the slow onset of drug effects and variations in patient compliance, monotherapy remains somewhat inadequate, necessitating further improvements in treatment methods. Recently, platelet‐rich plasma (PRP) has become a prominent topic in the treatment of hair loss and hair transplantation. PRP refers to autologous plasma that, after centrifugation, contains a higher‐than‐baseline concentration of platelets. It was first described in the 1970s in the context of hematology [7]. Subsequently, in orthopedics and sports medicine, PRP, because of its high concentration of growth factors, has been shown to stimulate soft tissue and joint healing [8]. Since the 1990s, PRP has been used in various medical fields, such as ophthalmology, oral and maxillofacial surgery, cardiovascular surgery, obstetrics and gynecology, and urology, to promote wound healing [9]. Since 2006, researches into PRP's role in stimulating hair growth have been ongoing, and it has been widely applied in clinical settings by plastic surgeons and dermatologists worldwide [10]. However, most studies are not strictly comparative clinical studies and differ from current treatment methods. Therefore, we believe there is considerable potential for optimizing PRP research for the treatment of AGA. On the basis of this, we conducted a prospective, comparative clinical study to better elucidate the role of PRP in hair transplantation.

## 2 Patients and Methods

This study adhered to the Declaration of Helsinki and received approval from the Ethics Committee. All patients provided informed consent. From August 2019 to December 2022, a total of 30 patients were included in the study. The inclusion criteria were as follows: (1) male patients with AGA, classified as Norwood–Hamilton grade 2–6; (2) female patients with AGA, classified as Ludwig grade 1–3; (3) patients aged between 20 and 50 years; and (4) signed informed consent. The exclusion criteria included the following: (1) presence of thyroid disorders, hematologic diseases, or scarring alopecia; (2) prior treatment for AGA; and (3) withdrawal from the study. We utilized a random number table to randomly assign the 30 enrolled patients into two groups, with 15 patients in the experimental group and 15 in the control group. Patients in all groups received oral spironolactone or finasteride combined with topical minoxidil treatment pre‐ and postoperatively. Patients in the experimental group underwent PRP injections during hair transplantation, whereas patients in the control group did not receive PRP for the transplanted hair.

### 2.1 Surgical Technique

We used follicle unit extraction (FUE) technique for hair transplantation. The day before the procedure, patients were given a haircut to keep the donor area hair at approximately 1 mm for better observation. The patient was positioned prone for disinfection with iodine tincture. Anesthesia was administered using a mixture of 5 g/L lidocaine, 1.875 g/L bupivacaine, 10 mL normal saline, and 20 mL of an epinephrine solution (1:100 000) for local anesthesia of the donor area. After anesthesia, a 1.0 mm diameter follicle‐specific extraction tool was used to separate the follicles and surrounding tissue to a depth of approximately 5–8 mm. During follicle separation, the procedure was conducted in the direction of hair growth to minimize follicle breakage. After extraction, the follicles were stored in ice‐cold saline at 0°C–4°C. The recipient area of the scalp was marked and anesthetized. Depending on the thickness of the follicles, either a 20G or 21G needle was used to create punctures in the direction of the original hair growth. The follicles were then implanted by holding the upper part with implantation tweezers and aligning them with the puncture slots, with puncturing and implantation occurring simultaneously. After the surgery, the wound was cleaned with saline, and the donor area was lightly compressed and bandaged with gauze.

### 2.2 PRP Preparation and Injection

**FIGURE 1:**
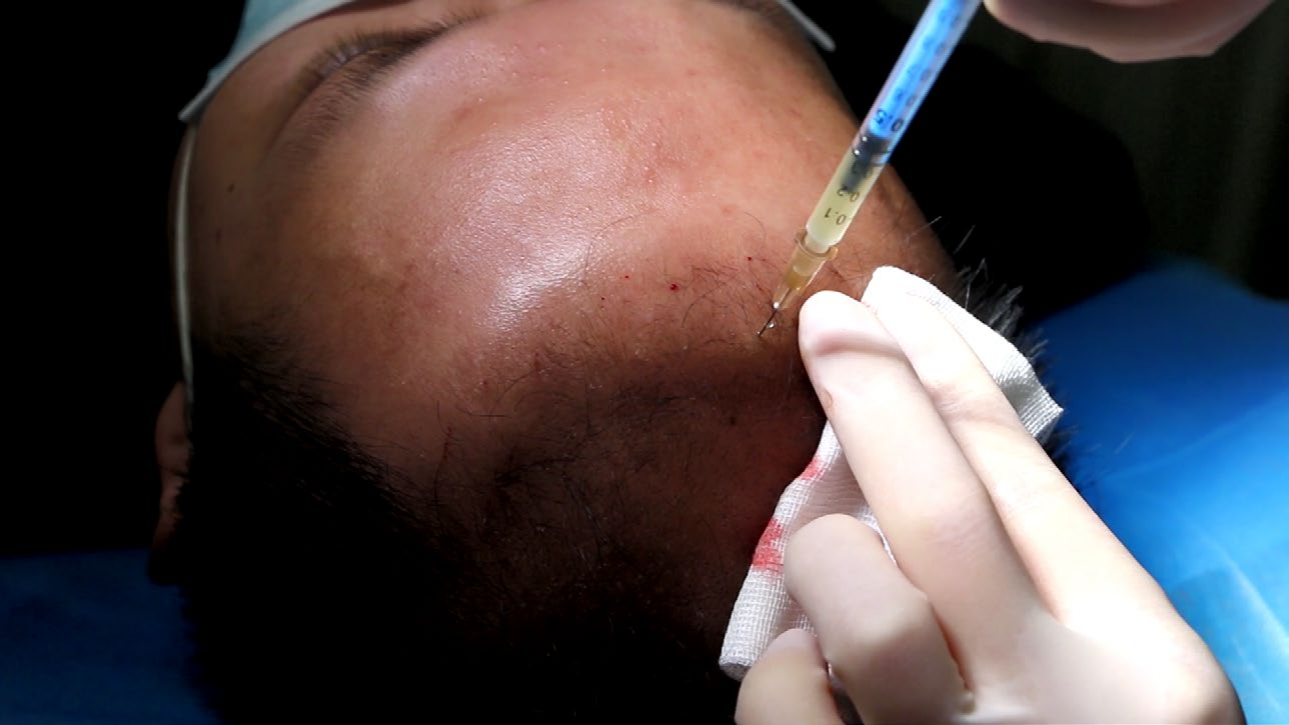
Injection of PRP before hair transplantation.

Before the FUE procedure, approximately 300–500 mL of venous blood was drawn from the patient's cubital fossa and injected into a vacuum blood collection tube containing sodium citrate, maintaining an anticoagulant‐to‐whole‐blood ratio of approximately 1 mL of anticoagulant to 10 mL of whole blood. The collected blood was gently mixed to ensure proper anticoagulant and blood integration. The blood is then centrifuged at a slow speed (soft spin, 101G) for 5 minutes to prevent platelet aggregation, ensuring they remain suspended in the supernatant. The platelet‐rich supernatant was transferred to another sterile tube and centrifuged at a higher speed (hard spin, 280 *g*) for 5 min to obtain PRP at the bottom of the tube. Approximately 50 mL of PRP was obtained for each patient in the experimental group, with the final PRP concentration being about five times the patient's own platelet count. The PRP was then placed in a 1 mL‐syringe, connected to a 32G needle and injected into the dermal layer of the scalp approximately every 1 cm. Each patient in the experimental group received about 15 mL of PRP before follicle extraction, and the remaining PRP was stored frozen for injection at 1 and 2 months postsurgery (Figure 1).

### 2.3 Postoperative Medication

On the first postoperative day, patients were instructed to clean the surgical area with saline at the hospital outpatient clinic. For the subsequent 3 weeks, they were to perform daily self‐cleaning and apply saline spray to the affected area every 2 hours to maintain local moisture. To prevent infection, antibiotics were prescribed for the first 3 days postsurgery. Additionally, during the peak swelling phase in the first 3 days, oral corticosteroids (dexamethasone, 3 mg once daily) were recommended. Recombinant fibroblast growth factor (FGF) gel (Zhuhai Yisheng Biopharmaceutical Co. Ltd.) was applied twice daily for 3 weeks.

Male patients were required to take finasteride on the day of surgery and continue postoperatively, although minoxidil was sprayed on the affected area. Minoxidil was applied once daily for the first week, followed by twice daily thereafter (once in the morning and once in the evening). Typically, medication can be gradually discontinued after 1–2 years of use. Finasteride was administered orally once daily at a dose of 1 mg for 2 years; thereafter, the dosage can be reduced to 0.5 mg daily for lifelong use. Female patients were required to take spironolactone at a dosage of 20 mg twice daily for 1–2 years, with a gradual reduction leading to discontinuation thereafter. The application method for minoxidil in female patients was the same as that for males.

### 2.4 Assessment Item

The indicators we need to evaluate include the following: (1) baseline characteristics of both groups such as gender, age, Norwood–Hamilton grade (for assessing male hair loss, with seven levels), Ludwig grade (for assessing female hair loss, with three levels), and platelet count; (2) the number of follicles transplanted during the procedure and the survival rate of these follicles; (3) the rate of hair growth posttransplant (measured by the time taken for transplanted follicles to begin growing) and hair strength (assessed through a traction test, where losing fewer than three hairs per pull is considered negative, and losing three or more hairs is considered positive); and (4) the incidence of postoperative complications.

### 2.5 Statistical Analysis

We used SPSS 26.0 (IBM Statistics for Windows, IBM Corp., Armonk, NY, USA) for data analysis. Data for both groups were expressed as means or medians, and differences were analyzed using the Kruskal–Wallis *H* test and chi‐squared test. A *p* value of < 0.05 was considered indicative of a significant difference.

## 3 Results

The average age of patients in the experimental group was 31.53 years, including 11 males and four females. In the control group, the average age was 32.8 years, with 13 males and two females. Preoperative hair loss was assessed using the Norwood–Hamilton grade for males and the Ludwig grade for females. The median Norwood–Hamilton grade for males in both the experimental and control groups was 4. The Ludwig grades for females in the experimental and control groups were 1 and 2, respectively. Preoperative platelet counts were also recorded, with mean values of 206.53 × 10^9^/L and 195.67 × 10^9^/L for the experimental and control groups, respectively. There were no significant differences in baseline characteristics between the two groups (Table 1).

**TABLE 1:** Patients baseline characteristics.

	PRP group	Control group
Age, years	31.53 ± 4.89	32.80 ± 5.60
Gender	11M, 4F	13M, 2F
Norwood–Hamilton grade	4 (3, 5)	4 (3, 4.5)
Ludwig grade	1 (1.5, 2.75)	2 (2.5, 3)
PLT × 10^9^/L	206.53 ± 79.53	195.67 ± 67.95

*Note:* The Norwood–Hamilton and Ludwig grades are represented by the median and interquartile range, whereas age and platelet count are represented by the mean ± SD.

Abbreviations: M: male, F: female.

The average number of transplanted follicular units was 1001.33 in the experimental group and 1087.33 in the control group, with no significant difference. The follicle survival rates at 3 months postsurgery were 88.42% for the experimental group and 78.24% for the control group (*p* = 0.001), indicating a significant difference. At 6 months postsurgery, the survival rates were 82.17% and 74.01% for the experimental and control groups, respectively (*p* = 0.002), showing a significant difference. We also recorded the time to initial hair growth posttransplant, with an average of 17.67 days for the experimental group and 20.07 days for the control group. For the strength of newly grown hair, traction tests were conducted at 3 months postsurgery. In the experimental group, 13 patients were negative and two were positive. In the control group, eight patients were negative and seven were positive. The analysis showed a significant difference in hair strength between the two groups (*p* = 0.046) (Table 2).

**TABLE 2:** Comparison of the number of follicle units' transplants, follicle survival rate, hair growth duration, and hair strength.

	PRP group	Control group	*p*
No. of transplanted follicle units	1001.33 ± 304.86	1087.33 ± 242.41	0.389
Survival rate %			
3 M postoperative	88.42 ± 6.21	78.24 ± 8.31	0.001*
6 M postoperative	82.17 ± 4.14	74.01 ± 5.25	0.002*
Growth duration	17.67 ± 2.23	20.07 ± 2.82	0.015*
Hair strength	13N, 2P	8N, 7P	0.046*

*Note:* All data are represented by mean ± SD.

Abbreviations: M: months, N: negative, P: positive.

* *p* < 0.05.

Postoperative complications were recorded for both groups, including bruising, pain, and hair curling. Additionally, one patient in the experimental group experienced transient itching at the surgical site, and one patient in the control group developed folliculitis because of inadequate postoperative self‐care (Table 3).

**TABLE 3:** Postoperative complications.

	PRP group	Control group
Hematoma	3	2
Epifolliculitis	0	1
Surgical area pain	2	1
Surgical area pruritus	1	0
Hair curl	2	3

## 4 Cases Report

### 4.1 Case 1

**FIGURE 2:**
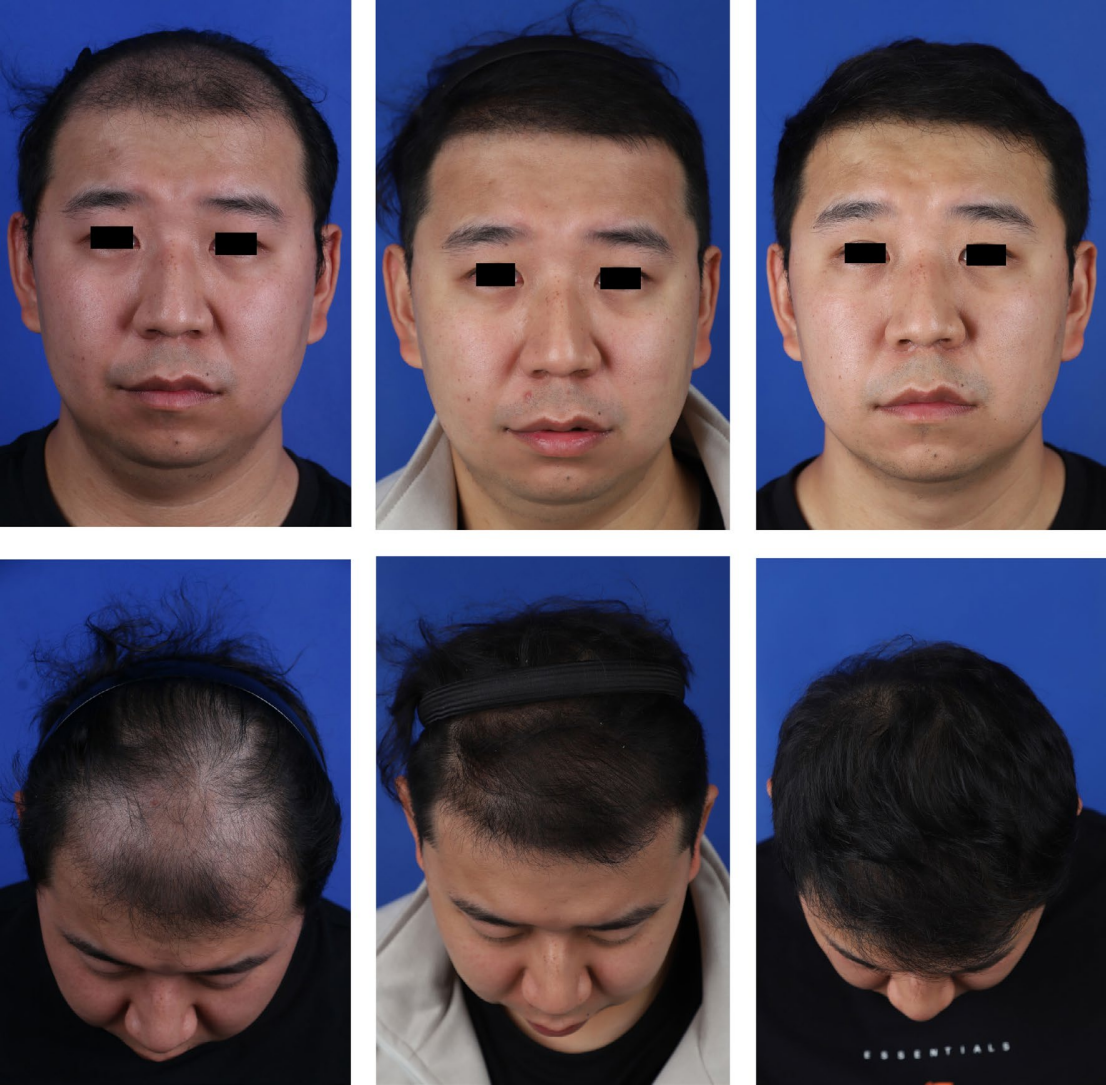
Images of the experimental group patient receiving PRP injection combined with FUE treatment: pretreatment, 3 months posttreatment, and 6 months posttreatment. Postoperative observation: The patient exhibits notably dense hair growth, indicating excellent treatment results.

**FIGURE 3:**
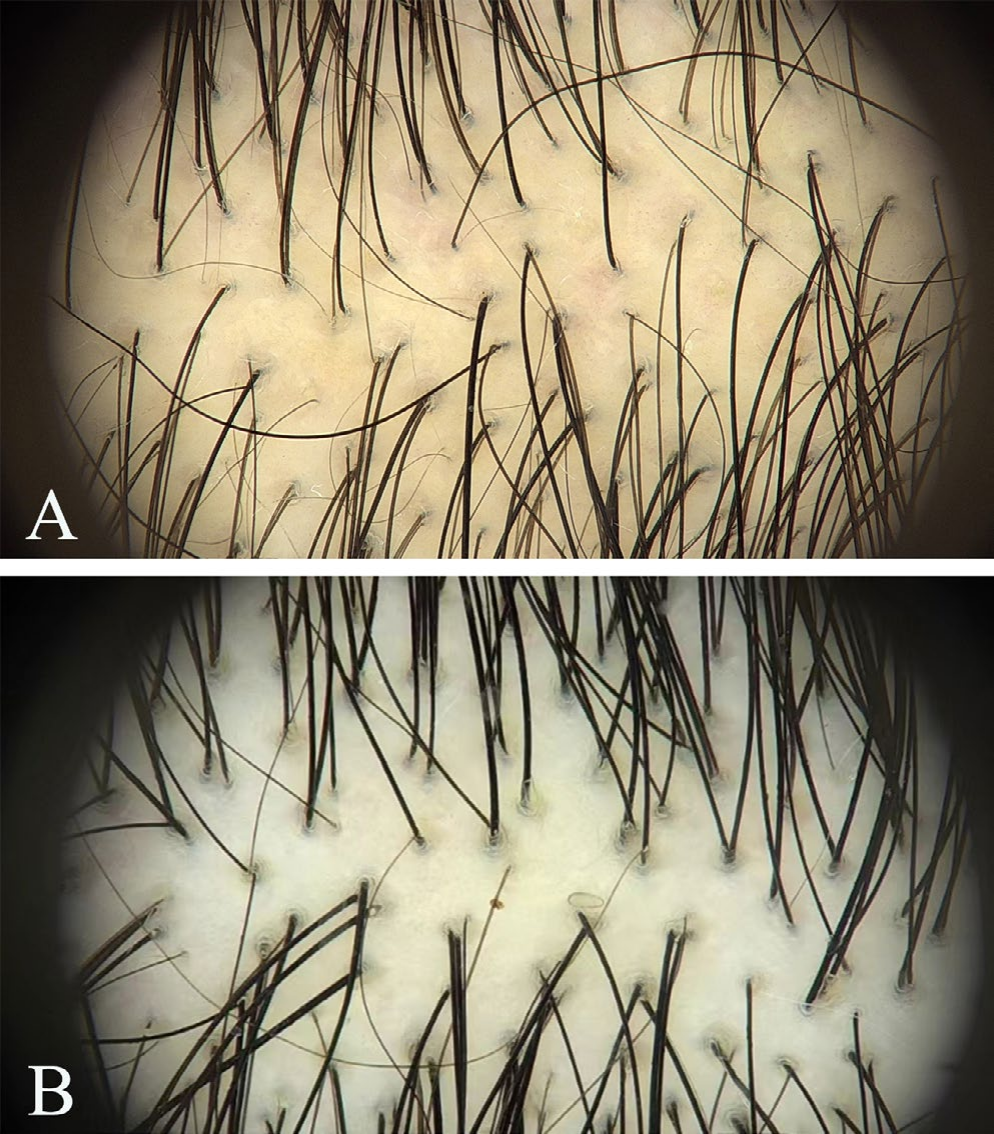
Microscopic images of hair from the experimental group patients before treatment (A) and at 6 months posttreatment (B) show a significant increase in hair growth after surgery.

The experimental group patient was a 34‐year‐old male who sought treatment because of gradual thinning of hair on the crown and a slight recession of the hairline, with a Norwood–Hamilton grade of 3 and a hair loss area of about 40 cm^2^. After evaluation, we diagnosed him with AGA. During the procedure, 950 follicular units were transplanted, and three PRP injections were administered at the time of the surgery, and at 1 and 2 months postsurgery. The patient was prescribed oral finasteride and topical minoxidil postoperatively. The follicle survival rates at 3 and 6 months were 85.5% and 81.0%, respectively. The patient was very satisfied with the treatment outcome (Figures 2 and 3).

### 4.2 Case 2

**FIGURE 4:**
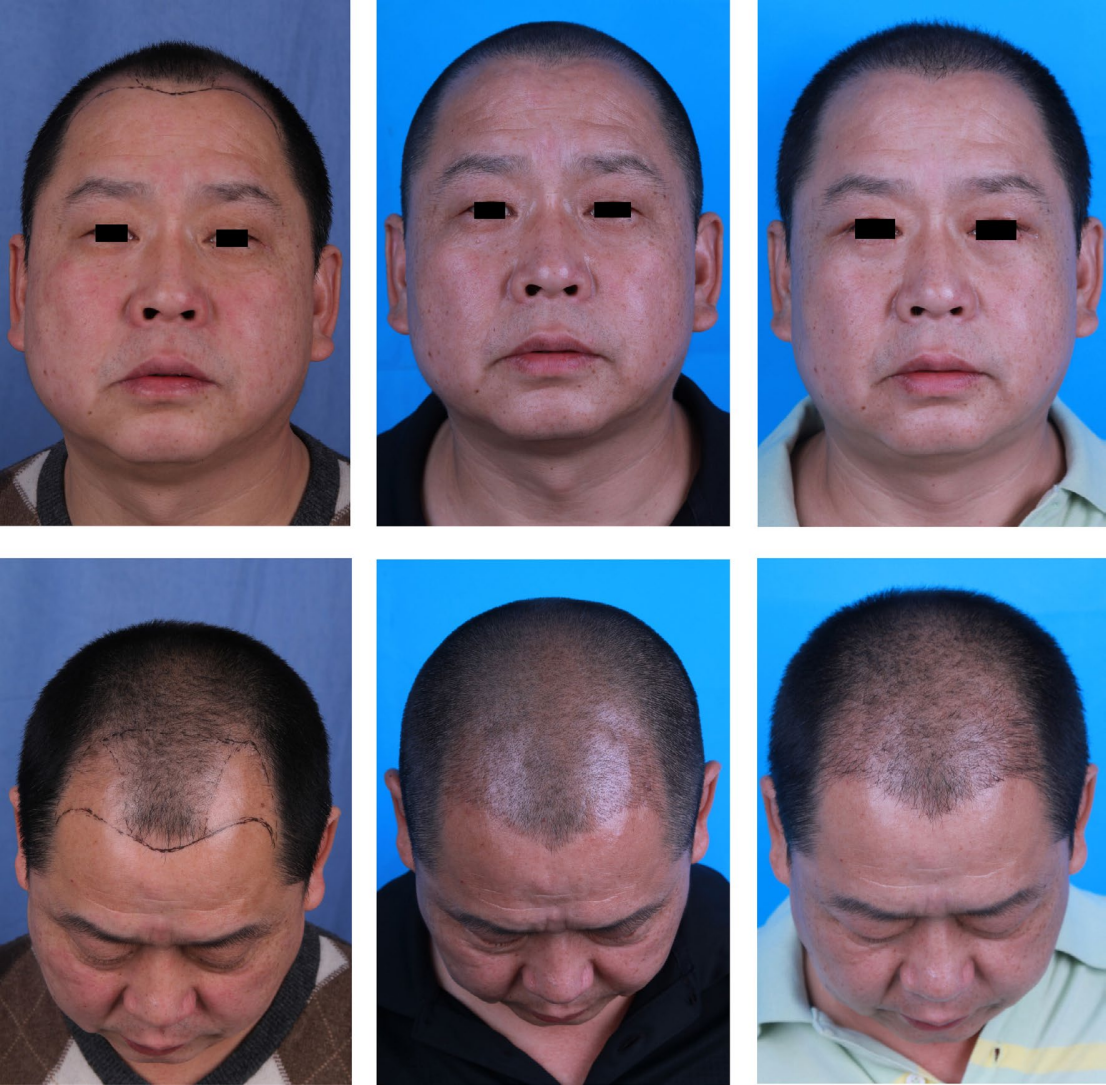
Images of the control group patient undergoing FUE treatment alone: pretreatment, 3 months posttreatment, and 6 months posttreatment. Postoperative observation: Transplanted hair follicles exhibit some hair growth; however, certain areas appear sparse.

The control group patient was a 42‐year‐old male who sought treatment at our hospital because of persistent hair loss for over 10 years. His frontal and lateral hairlines had retreated approximately 3.5 cm, and he exhibited significant thinning of hair on the crown, with a Norwood–Hamilton grade of 4 and a hair loss area of about 110 cm^2^. He was subsequently diagnosed with AGA. During the procedure, 1300 follicular units were transplanted. The patient was prescribed oral finasteride and topical minoxidil postoperatively. The follicle survival rates at 3 and 6 months were 79.6% and 72.5%, respectively. The patient expressed general satisfaction with the treatment outcome, although we believe there is still room for improvement (Figure 4).

## 5 Discussion

AGA is a type of hair loss disease primarily mediated by androgens and genetic factors, characterized by progressive hair shedding and gradual follicular atrophy. Approximately 30% of men over the age of 30 are affected by AGA, with the prevalence rising to around 50% in men over 50 [11]. Additionally, a portion of women may also be affected by this condition. For diagnosing AGA, a thorough medical history and physical examination are crucial. Patients should also undergo comprehensive laboratory tests to rule out other causes of hair loss, such as anemia, nutritional deficiencies, and thyroid dysfunction. Laboratory tests typically include a complete blood count and measurements of serum iron, ferritin, total iron‐binding capacity, and folate levels. Thyroid function tests include T3, T4, thyroid‐stimulating hormone, and antithyroid peroxidase antibodies. Other endocrine tests include measurements of testosterone and other hormones. Diagnosis markers in autoimmune disease, such as antinuclear antibodies, may also be assessed [12]. Once diagnosed with AGA, patients should promptly start treatment with minoxidil and finasteride/spironolactone. When these treatments are insufficient, patients may prefer invasive options such as scalp flaps, tissue expansion, follicular unit transplantation, and follicular unit extraction. Currently, FUE technology is widely used because of its minimal trauma, fast healing, and absence of linear scarring.

PRP, which is autologous plasma with a higher platelet concentration than baseline after centrifugation, has become one of the most popular treatments for hair loss, particularly AGA, in recent years. When platelets in PRP are activated, their α‐granules release various growth factors, including platelet‐derived growth factor, transforming growth factor‐beta, FGF‐2, vascular endothelial growth factor, epidermal growth factor, insulin‐like growth factor‐1, and glial cell–derived neurotrophic factor [13]. Thus, PRP improves the microenvironment of the transplanted area by promoting cell growth and differentiation, inhibiting apoptosis, and enhancing the formation of new blood vessels, making the transplant area more conducive to new hair growth. It also provides favorable conditions for dormant follicles, reactivating them and promoting new hair growth within 3 months. Our research results confirm that PRP not only increases the survival rate of transplanted follicles but also improves the speed of hair growth and hair strength.

Although PRP has demonstrated promising adjunctive therapeutic effects, several uncertainties still limit its broader application. Firstly, there is no consensus among researchers regarding the optimal platelet concentration in PRP. Marx et al. [14] suggest that a platelet concentration exceeding 1 000 000 platelets/μL per 5 mL of plasma, or 2–7 times an individual's baseline concentration, yields better results. In contrast, Graziani et al. [15] recommend that the optimal concentration should be 2.5 times the baseline, as exceeding this level might inhibit effectiveness.

Secondly, there is a lack of standardized protocols for the preparation and composition of PRP [16]. PRP preparation can generally be classified into open and closed methods. The open method involves preparing PRP in an uncontrolled environment, exposing the blood to external conditions during processing. This method, which typically involves two rounds of centrifugation to extract PRP, is favored in most dermatological practices because of its lower cost and higher platelet yield. Conversely, the closed method avoids contact with external environments during blood preparation and primarily utilizes commercial kits along with compatible centrifuges. The most common kits employ gel separation technology, containing 1–2 mL of thixotropic polymer gel in blood collection tubes. This gel has a lower specific gravity than red and white blood cells, although approximately 15 different commercial kits are available [17].

In addition to differences in preparation methods, the composition of PRP can also vary. Some researchers argue that PRP should exclusively contain platelets, termed pure platelet‐rich plasma (P‐PRP). Others contend that PRP may include white blood cells or fibrin, resulting in formulations such as leukocyte‐rich platelet plasma (L‐PRP) and pure platelet‐rich fibrin [18]. Thus, the primary limitations of current clinical research on PRP lie in the uncertainty and inconsistency regarding optimal concentration, composition, and preparation methods. This represents one of the limitations of our study. Future research on PRP should focus on determining its specific therapeutic effects through large‐scale, multicenter, prospective randomized studies.

Although some researchers have investigated PRP in conjunction with hair transplantation, indicating its advantages, we believe these studies still have shortcomings. Firstly, some studies have not strictly adhered to comparative research standards. Secondly, certain studies have not included the concurrent use of minoxidil and finasteride/spironolactone. Although this approach may mitigate the influence of other medications on PRP and avoid confounding results, we argue that for patients, attempting a combination of medications with PRP to maximize hair restoration following FUE is significantly meaningful.

## 6 Conclusion

Our prospective, comparative clinical study demonstrated that patients in the experimental group, who received PRP in combination with minoxidil and finasteride/spironolactone along with FUE, exhibited significantly better follicle survival rates, hair growth rates, and hair strength than in the control group, achieving excellent treatment outcomes.

## Author Contributions

P.X., E.D., and W.D. performed the research. Q.Y. and X.F. supervised the research study. X.Z. and L.G. analyzed the data. P.X. and L.G. wrote the paper.

## Conflicts of Interest

The authors declare no conflicts of interest.

## Data Availability

The data that support the findings of this study are available from the corresponding author upon reasonable request.
